# Bacteriostatic and Bactericidal Effect of Tigecycline on *Leptospira* spp.

**DOI:** 10.3390/antibiotics9080467

**Published:** 2020-07-30

**Authors:** Fabrizio Bertelloni, Giovanni Cilia, Filippo Fratini

**Affiliations:** Department of Veterinary Sciences, University of Pisa, Viale delle Piagge 2, 56124 Pisa, Italy; fabrizio.bertelloni@unipi.it (F.B.); filippo.fratini@unipi.it (F.F.)

**Keywords:** *Leptospira*, minimum inhibitory concentration (MIC), microdilutions method, tigecycline, antibiotic susceptibility, leptospirosis

## Abstract

Tigecycline is a relatively new antimicrobial, belonging to glycylcyclines with antimicrobial activity against a large spectrum of bacteria. Very few data are available on its effect on *Leptospira* spp., which consist in a bacteriostatic mechanism. The aim of this investigation was to evaluate the bacteriostatic and bactericidal effect of tigecycline on reference *Leptospira* strains belonging to 16 serovars. Minimum inhibitory concentration (MIC) and minimum bactericidal concentration (MBC) were determined through the microdilutions method, and tetracycline was used as the control. Results showed that tigecycline had higher MIC and MBC values than tetracycline. Obtained MIC values were between 4 and 32 µg/mL, while MBC values between 16 and >128 µg/mL. Patoc (MIC: 4 µg/mL; MBC: 16 µg/mL) resulted in the most susceptible serovar, while the most resistant were Bataviae (MIC: 32 µg/mL; MBC: 64 µg/mL), Bratislava (MIC: 8 µg/mL; MBC 128 µg/mL), and Tarassovi (MIC: 8 µg/mL; MBC: >128 µg/mL). This is the first investigation focused on the effect of tigecycline against *Leptospira* spp. reference strains. Since tigecycline is used as a treatment for bacteremia and urinary tract disease, and these symptoms could be linked to *Leptospira* infection, the possibility of using this antibiotic as a treatment for leptospirosis should be evaluated. Further studies are needed to explore the possibility to use tigecycline for in vivo application against *Leptospira*.

## 1. Introduction

Leptospirosis is a zoonotic re-emerging disease caused by spirochetal Gram-negative bacteria belonging to genus *Leptospira* [1]. Recently, more than 260 antigenically distinct serovars of *Leptospira* spp. were isolated and identified, and the genus *Leptospira* was sub-grouped, based on it DNA relatedness to pathogenic, intermediate, and saprophytic species on the basis of the different levels of virulence exhibited for animals and humans. In detail, pathogenic *Leptospira* causes mild to severe infection, intermediate *Leptospira* may potentially be a pathogen and is responsible for mild infections, while saprophytic *Leptospira* spreads in environments and is a nonpathogen [2,3,4]. Recent studies highlighted the important role of intermediate and saprophytic *Leptospira* in the epidemiology of leptospirosis, because these can share habitats and could give rise to recombination events with pathogenic serovars [5,6,7].

Leptospirosis is diffused worldwide and occurs in tropical, subtropical, and temperate zones, representing public health problems, since its epidemiology involves humans and domestic and wild animals, which can be maintenance or accidental hosts [1,8,9]. Maintenance hosts, generally do not develop symptoms referable to disease and they represent important renal carriers, contributing to maintaining and sharing the infection, shedding *Leptospira* with urine in the environment. The accidental contact with *Leptospira* colonized urine represents the primary cause of incidental infection, producing clinical diseases [1,4]. For these reasons, *Leptospira* epidemiology is strictly connected to maintenance host species [10]. Therefore, specific *Leptospira* serovars are related to specific animal species, which involves specific maintenance hosts. Generally, for example, rodents are associated with Icterohaemorrhagiae and Ballum serogroups [11,12,13], swine with Pomona and Tarassovi serogroups [14,15,16,17,18], horse with Bratislava serogroups [19,20], and bovine and ovine with Sejroe serogroups [21,22].

Leptospirosis by pathogenic serovars can cause mild to severe infections in both humans and animals. Most cases are mild and they resolve spontaneously. [23]. Conventionally, the treatment for acute and severe leptospirosis consists of strong antimicrobial therapy with tetracycline, penicillin, or doxycycline [1,23]. Generally, molecules belonging to the tetracycline class are those mainly used in veterinary and human practices [1,23,24,25].

Tigecycline is a relatively new antimicrobial, belonging to glycylcyclines, which derive from tetracyclines [26].

Tigecycline showed antimicrobial effects in vitro against many Gram-positive and Gram-negative bacteria, including multi-drug resistant strains (methicillin resistant *Staphylococcus aureus*, extended spectrum beta-lactamase (ESBL)-producing Enterobacteriaceae, multi-drug resistant *Acinetobacter baumanii*, and vancomycin-resistant *Enterococcus* spp.) [27,28]. The action of tigecycline consists of a bacteriostatic mechanism, which relies on the binding of 30 s ribosomal subunits of bacteria and blocking the entry of transfer of RNA [26].

Studies on the antimicrobial effects on *Leptospira* isolates or reference cultures are scarce, probably due to problems in the determination of antibiotic susceptibility [1,23]. A standardized microdilution method has been developed to accurately investigate the *Leptospira* antibiotic susceptibility, resolving the difficulties related to preventing the contamination of the medium, evaluating the bacterial growth, and reproducibility of the assay [29,30,31].

The aim of this investigation was to evaluate, through the microdilutions method, the bacteriostatic and bactericidal effects of tigecycline on reference *Leptospira* strains belonging to 16 serovars.

## 2. Results

Table 1 shows the minimum inhibitory concentration (MIC) and minimum bactericidal concentration (MBC) values of tetracycline and tigecycline for different strains of *Leptospira* spp.

For tetracycline, MIC values were between 0.5 and 2.0 µg/mL, while MBC values were between 16 and 64 µg/mL. Canicola, Autumnalis, and Hebdomadis resulted in the most susceptible serovars, with MIC values of 0.5 µg/mL, while MBC values were between 16 and 32 µg/mL. On the other hand, Tarassovi and Ballum resulted in the most resistant serovars with MIC and MBC values of 2 and 64 µg/mL, respectively.

Concerning tigecycline, obtained MIC values were between 4 and 32 µg/mL, while MBC values between 16 and >128 µg/mL. Patoc resulted in the most susceptible serovar, with MIC and MBC values of 4 and 16 µg/mL, respectively. Additionally, the most resistant serovars were Bataviae (32 µg/mL for MIC and 64 µg/mL for MBC), Bratislava (8 µg/mL and 128 µg/mL), and Tarassovi (8 µg/mL and >128 µg/mL). 

## 3. Discussion

Treatments for *Leptospira* infections are based on the use of antibiotics belonging to a few specific molecules, generally tetracyclines. These antibiotics are conventionally used in medicine, thanks to no antimicrobial resistant events highlighted for *Leptospira.* The knowledge of antibiotic treatments against leptospirosis is strictly connected to in vivo clinical evaluation, usually in patients with severe and acute *Leptospira* infections [1,4,23]. Very few studies explored the efficacy and mechanisms of the action of new chemotherapeutic molecules [29,30,31]. This is the first investigation showing the in vitro effect of tigecycline against *Leptospira* spp.

The obtained results were consistent with other data available in literature, according to which tetracycline MIC values for *Leptospira* reference strains or isolates are, on average, between 0.25 and 6.25 µg/mL [29,30,31,32,33,34]. Considering that the results obtained for tetracycline were very similar to those reported in literature, it is correct to suppose that the results for MIC and MBC for tigecycline were also similar, not under- or overestimated. Since tetracycline and tigecycline belong to the same antibiotic class, it is reasonable to exclude any causes of strain attenuation due to the passages for culture maintenance, mutation, or acquisition of genes for antibiotic resistance.

Results showed that tigecycline had higher MIC and MBC values than tetracycline, used as the control. To the best of the authors’ knowledge, no studies have focused on the tigecycline effect on *Leptospira*, excluding one in vivo assay. An administration of 5 mg/kg/day of tigecycline for 5 days was useful to reduce the presence of spirochetes in the blood of hamsters experimentally infected with 10^5^ *Leptospira interrogans* serovar Canicola [35].

Considering spirochetes sensu latu, tigecycline showed inhibitory effects on *Borrelia* spp. *Borrelia burgdorferi* was susceptible to tigecycline at concentrations between 0.006 and 6 µg/mL [36,37,38,39]. Additionally, for *Borrelia bavariensis, B. garinii, B. afzelii, B. spielmanii, B. valaisiana*, and *B. lusitaniae*, MIC values between 0.012 and 0.5 µg/mL have been reported [38,39]. Moreover, tigecycline MBC values for *Borrelia* spp. were between 4 and 16 µg/mL [39]. Results on the *Borrelia* spp. tigecycline effect were similar to those obtained in this investigation against *Leptospira*.

Although the tigecycline effect was investigated only in one in vivo study on *Leptospira* and in other studies on different spirochetes species, this investigation highlighted the bacteriostatic and bactericidal effect on *Leptospira* spp. strains of this antimicrobial molecule. Since this is the first investigation, further studies should be carried out to understand the antimicrobial effects against *Leptospira,* using reference strains and isolates, and to determine its potential employment for leptospirosis treatment or antimicrobial resistance. Moreover, further genomic studies are required to elucidate the molecular mechanisms responsible to elucidate the *Leptospira* antimicrobial susceptibility or resistance. *Leptospira* species don’t have the ability to acquire genes for antimicrobial resistance during horizontal transfer and, if present, it is probably limited [24,31]. In humans, leptospirosis is a dead-end infection, where human-to-human transmission is very rare, considering that the infection is also usually monomicrobial, horizontal resistance gene acquisition from other bacterial species, as well as in the environment, very difficult [24]. Finally, the major causes of *Leptospira* antibiotic resistance seem to be related to spontaneous target gene mutations, as was in vitro evaluated for spontaneous mutation of the target gene *16S* rRNA and *rpsL* for the development of spectinomycin and streptomycin resistance, respectively [40,41].

## 4. Materials and Methods 

### 4.1. Leptospira spp. Strains

In this investigation, the following references of *Leptospira* strains were employed: *Leptospira interrogans* serovar Icterohaemorrhagiae (serogroup Icterohaemorrhagiae, strain Bianchi); *L. interrogans* serovar Canicola (serogroup Canicola, strain Alarik); *L. interrogans* serovar Pomona (serogroup Pomona, strain Mezzano); *L. kirschneri* serovar Grippotyphosa (serogroup Grippotyphosa, strain Moskva V); *L. borgpetersenii* serovar Tarassovi (serogroup Tarassovi, strain Mitis Johnson); *L. interrogans* serovar Bratislava (serogroup Australis, strain Riccio 2); *L. interrogans* serovar Hardjo (serogroup Sejroe, serovar Hardjoprajitno); *L. borgpetersenii* serovar Ballum (serogroup Ballum, strain Mus 127); *L. interrogans* serovar Copenhageni (serogroup Icterohaemorrhagiae, strain Wijmberg); *L. interrogans* serovar Bataviae (serogroup Bataviae, strain Pavia); *L. interrogans* serovar Zanoni (serogroup Pyrogenes, strain Zanoni); *L. borgpetersenii* serovar Poi (serogroup Javanica, strain Poi); *L. interrogans* serovar Lora (serogroup Australis, strain Riccio 37); *L. interrogans* serovar Autumnalis (serogroup Autumnalis, strain Akiyami A); *L. interrogans* serovar Hebdomadis (serogroup Hebdomadis, strain Hebdomadis); and *Leptospira biflexa* serovar Patoc (serogroup Semaranga, strain Patoc I).

Each strain was maintained in pure culture in the Ellinghausen–McCullough–Johnson–Harris (EMJH) medium, sub-cultured, and checked weekly for purity and viability.

### 4.2. Antibiotics

Tigecycline was purchased from Sigma–Aldrich (St. Louis, MO, USA), while tetracycline was purchased from Carlo Erba (Cornaredo, MI, Italy). Tetracycline was employed as the control to compare the results of tigecycline effects.

### 4.3. Minimal Inhibitory Concentration (MIC) and Minimal Bactericidal Concentration (MBC)

The MIC determination was performed by the broth microdilution method, described by Liegeon et al. [31]. *Leptospira* cultures were quantified with spectrophotometry using optical density at 420 nm (Multiskan™ FC Microplate Photometer; Thermo Fisher Scientific, Haverhill, MA, USA) to reach a turbidity of 0.5 of the McFarland standard, which corresponds to approximately 1.5 × 10^8^ cfu/mL [42,43]. In each well of 96-well plates, two-fold serial dilutions were performed in EMJH media, ranging from 128 to 0.25 μg/mL and from 64 to 0.125 μg/mL for tigecycline and tetracycline, respectively. The final volume in each well was 100 μL, including 5 μL of standardized strain. Two microplates were prepared for each strain: one to determine the MIC and the other for the MBC. They were incubated for 3 days at 30 °C, then 20 μL of resazurin sodium salt (Alfa Aesar, Thermo Fisher Scientific, Haverhill, MA, USA), diluted 1:30 in EMJH medium, was added to each well. The plates were incubated at 30 °C for another 2 days. A change in color from blue to pink indicated *Leptospira* growth. The MIC value was reported as the lowest concentration able to prevent a color change. 

MBC was determined by plating 10 μL from each well (starting from the MIC value to higher antibiotic concentrations) in 1.5% EMJH agar. Plates were incubated at 30 °C for 4 days. The MBC value was reported as the lowest concentration able to prevent bacterial growth.

All tests were performed in triplicate and the results were expressed as the mode.

## 5. Conclusions

Tigecycline administration is usually employed in human patients for treatment of bacteria that causes pneumoniae and diabetic foot infections, but also for urinary tract infections and bacteremia [44,45]. Considering that bacteremia and urinary tract diseases could be symptoms compatible with *Leptospira* infection, the possibility of using this antibiotic as a treatment for leptospirosis should be evaluated. Data obtained in this in vitro investigation seem to be encouraging, and further studies are auspicial to explore the possibility of using tigecycline for in vivo application against *Leptospira*.

## Figures and Tables

**Table 1:** Determination of minimum inhibitory concentration (MIC) and minimum bactericidal concentration (MBC) for tetracycline and tigecycline among the 16 investigated *Leptospira* spp. strains. Results are expressed as mode and values reported in µg/mL.

*Leptospira* Serovar	Tetracycline		Tigecycline	
	MIC	MBC	MIC	MBC
Icterohaemorrhagiae	2	16	8	64
Canicola	0.5	32	4	32
Pomona	1	16	8	32
Tarassovi	2	64	8	>128
Grippotyphosa	1	16	8	64
Bratislava	1	32	8	128
Ballum	2	64	8	64
Hardjo	1	32	8	32
Copenhageni	2	32	8	32
Bataviae	2	32	32	64
Zanoni	1	32	4	32
Poi	2	16	8	32
Lora	2	32	16	32
Autumnalis	0.5	16	8	32
Hebdomadis	0.5	16	8	64
Patoc	2	8	4	16
